# Characterization of a novel brain barrier ex vivo insect-based P-glycoprotein screening model

**DOI:** 10.1002/prp2.50

**Published:** 2014-06-09

**Authors:** Olga Andersson, Liesbeth Badisco, Ane Håkansson Hansen, Steen Honoré Hansen, Karin Hellman, Peter Aadal Nielsen, Line Rørbæk Olsen, Rik Verdonck, N Joan Abbott, Jozef Vanden Broeck, Gunnar Andersson

**Affiliations:** 1 EntomoPharm, R&D Medicon Village, S-223 81, Lund, Sweden; 2 Department of Animal Physiology and Neurobiology, Katholieke Universiteit Leuven Leuven, Belgium; 3 Department of Pharmacy, Faculty of Health and Medical Sciences, University of Copenhagen DK-2100, Copenhagen, Denmark; 4 BBB Group, Institute of Pharmaceutical Science, King′s College London Franklin Wilkins Building, London, SE1 9NH, United Kingdom

**Keywords:** Brain–barrier permeability, efflux transporter, insect brain barrier, Pgp-transporter, screening model

## Abstract

In earlier studies insects were proposed as suitable models for vertebrate blood–brain barrier (BBB) permeability prediction and useful in early drug discovery. Here we provide transcriptome and functional data demonstrating the presence of a P-glycoprotein (Pgp) efflux transporter in the brain barrier of the desert locust (*Schistocerca gregaria*). In an in vivo study on the locust, we found an increased uptake of the two well-known Pgp substrates, rhodamine 123 and loperamide after co-administration with the Pgp inhibitors cyclosporine A or verapamil. Furthermore, ex vivo studies on isolated locust brains demonstrated differences in permeation of high and low permeability compounds. The vertebrate Pgp inhibitor verapamil did not affect the uptake of passively diffusing compounds but significantly increased the brain uptake of Pgp substrates in the ex vivo model. In addition, studies at 2°C and 30°C showed differences in brain uptake between Pgp-effluxed and passively diffusing compounds. The transcriptome data show a high degree of sequence identity of the locust Pgp transporter protein sequences to the human Pgp sequence (37%), as well as the presence of conserved domains. As in vertebrates, the locust brain–barrier function is morphologically confined to one specific cell layer and by using a whole-brain ex vivo drug exposure technique our locust model may retain the major cues that maintain and modulate the physiological function of the brain barrier. We show that the locust model has the potential to act as a robust and convenient model for assessing BBB permeability in early drug discovery.

## Introduction

The mammalian blood–brain barrier (BBB) is composed of capillary endothelial cells that control the entry of nutrients and xenobiotics to the brain and thus preserve homeostasis of the neural microenvironment, a prerequisite for reliable neural transmission and function (Abbott et al. 2006, 2010). However, the protection of brain function by the BBB restricts the permeation of drugs and results in a low brain target exposure concentration being a major challenge in the discovery of new drugs for central nervous system (CNS)-related diseases.

An important gate keeper in the vertebrate brain is the ATP-driven P-glycoprotein (Pgp) efflux transporter (multidrug resistance protein-1[MDR1], ATP-binding cassette [ABC] B1), localized to the endothelium forming the BBB. The efficiency of the Pgp transporter in restricting the uptake of therapeutic drugs has been demonstrated several times, for example by comparing brain uptake in Pgp knockout (KO) mice with uptake in wild type (WT) (Schinkel 1999; Doran et al. 2005). The promiscuity of the Pgp transporter enables it to efflux structurally unrelated hydrophobic amphipathic compounds, including therapeutic drugs. The Pgp-imposed restriction of drug permeation of the BBB has been a critical issue in the drug discovery process resulting in significant efforts to establish experimental models providing information on CNS barrier efflux of test compounds. Although comparison of brain:plasma ratio (B/P) in WT and Pgp KO mice generates solid Pgp information, this in vivo model is too time-consuming to be used as standard screening model in early drug discovery. Therefore, a number of cell-based in vitro models have been developed and proposed as useful tools in the drug discovery screening process and in the identification of Pgp substrates and inhibitors (Polli et al. 2001; Weiss et al. 2003; Mensch et al. 2009).

Two commonly employed systems are the renal cell lines LLC-PK1 (porcine kidney epithelial cells) and Madin-Darby canine kidney (MDCK). Both cell systems are easy to grow and MDCK, transfected with the human multidrug resistance gene MDR1 (MDR1-MDCK, MDR1-transfected MDCK)*,* is often used as an industrial screening model for identification of Pgp substrates (Feng et al. 2008). However, these cells are of epithelial origin and compared to barrier endothelial cells, epithelial cells display differences in morphology, tight junction organization, and transporter expression (Garberg et al. 2005; Cecchelli et al. 2007; Abbott et al. 2008; Liu et al. 2008). Furthermore, screening data from the MDCK model showed that more than 70% of the non-CNS drugs exhibited permeability above the level recommended for selection of CNS candidates. In addition, the MDR1-MDCK cells possessed Pgp transporter activity markedly greater than in vivo (Mahar Doan et al. 2002; Feng et al. 2008), making the prediction of the in vivo properties problematic.

In general, CNS drugs should be characterized by reasonable BBB permeability and low Pgp efflux while peripheral-acting drugs should either be poorly permeating or substrates for efflux transporters, for example Pgp or breast cancer-resistant proteins (BCRP). Pgp is the most intensively studied transporter and it is a major concern to the pharmaceutical industry (Liu et al. 2008). In a comprehensive study using Pgp (*mdr1a/b*) KO and WT mice, it was confirmed that the majority of the 32 most prescribed CNS drugs showed no or weak Pgp-mediated transport (Doran et al. 2005). From another study (Mahar Doan et al. 2002), using the in vitro MDR1-MDCK model, it was found that the majority of the non-CNS drugs exhibited a permeability rate that overlapped that of the CNS drugs, demonstrating much lower accuracy in this model compared to the in vivo KO/WT Pgp discrimination. These discrepancies most probably reflect higher paracellular permeability due to less functional tight junctions of the MDR1-MDCK cells but could also be influenced by the overexpression or background expression of Pgp transporter in transfected and WT cells (Taub et al. 2005; Kuteykin-Teplyakov et al. 2010). For this reason the need for models that have permeability and transporter characteristics more similar to those of the vertebrate BBB has been emphasized (Liu et al. 2008).

Recently we found, using a locust brain barrier model, that the Pgp inhibitor verapamil significantly increased uptake of the Pgp substrate quinidine into the locust brain (Nielsen et al. 2011). This observation was strongly supported by the documentation in the fruit fly (*Drosophila melanogaster, Dm*) of the mammalian Pgp transporter homologue (*Mdr65*) (Mayer et al. 2009). Here we have characterized the locust model in vivo by intrahemolymphic injection of the test compound and ex vivo under controlled test compound exposure conditions.

## Materials and Methods

### Animals

Desert locusts, *Schistocerca gregaria* (*Sg*), were obtained from a commercial animal breeder (Petra Aqua, Prague, Czech Republic). On arrival the locusts were housed under crowded conditions in an insect room and adapted to a 12:12 h dark/light cycle. The locusts were maintained in colonies at a local terrarium temperature of 30–34°C. The animals were fed Chinese cabbage and wheat bran ad libitum. All experiments were carried out on fifth instar locusts and 2–3 weeks after adult emergence.

### Chemicals

The following compounds were obtained from Sigma-Aldrich (Stockholm, Sweden): atenolol, carbamazepine (Cbz), loperamide, propranolol, rhodamine 123 (Rho123), quinidine, and verapamil. Cyclosporin A (CsA; Sandimmun, 50 mg/mL, Novartis, Tärby, Sweden) was purchased from the drug store, Apoteket AB (Lund, Sweden). All other chemicals were analytical reagent grade and were purchased from Sigma-Aldrich.

### Test solution preparation

Stock solutions were prepared by dissolving the test compounds in 100% dimethyl sulfoxide (DMSO). Final test solution concentrations were obtained by diluting stock solutions in 4-(2-hydroxyethyl)-1-piperazineethanesulfonic acid (HEPES)-buffered insect buffer with the following composition: NaCl 147 mmol/L, KCl 10 mmol/L, CaCl_2_ 4 mmol/L, NaOH 3 mmol/L, and HEPES 10 mmol/L (pH 7.2). For in vivo studies, Rho123 and loperamide were dissolved in H_2_O with 2.5% DMSO as final concentration and CsA (Sandimmun) diluted with saline. Verapamil was dissolved in insect buffer.

### In vivo experiments

Drugs were administrated into the hemolymph by inserting the needle (27G × ¾″) of a 100 *μ*L Hamilton syringe between two terga of the abdomen of the locust. 40 *μ*L of the test compound solution was injected into the hemolymph. The test compounds were dosed using the following concentrations: Rho123 at 1.25 mg/mL, loperamide at 2.5 mg/mL, and CsA at 1.2 mg/mL. After 15 min exposure at room temperature (20°C) 10 *μ*L hemolymph was sampled using Disposable Capillary Pipettes “end to end” and transferred to 40 *μ*L ice-cooled insect buffer in a 1.5 mL microcentrifuge tube (Eppendorf, Nordic, Hörsholm, Denmark). Hemolymph proteins were precipitated and further prepared for analysis as described below.

The brain of the treated animal was immediately dissected after hemolymph sampling by cutting off the frontal part of the head, through the esophagus. Following removal of the neural lamella (the outer connective tissue layer), the brains were washed in ice-cold insect buffer and transferred to 1.5 mL microcentrifuge tubes (Eppendorf) containing 50 *μ*L ice-cold insect buffer. The brains where further prepared for analysis as described below. (The size of the desert locust brain in males and females was determined as 1.60 ± 0.09 mg and 1.68 ± 0.16 mg, respectively [*n* = 12].)

The brain uptake of the test compound is related to the brain exposure but since the test compound concentration in the hemolymph may vary between individual animals, we base the comparisons on brain:hemolymph ratios rather than on absolute brain concentrations.

Clearance of test compounds from the hemolymph was measured by repeated sampling of 10 *μ*L hemolymph from the individual locust at 5, 15, 45, 120, and 360 min after intrahemolymph administration (20 *μ*L) of the test compound solution (20 *μ*mol/kg body weight). The 10 *μ*L hemolymph samples were added to microcentrifuge tubes (Eppendorf) containing 40 *μ*L ice-cold insect buffer. Hemolymph proteins were precipitated and further prepared for analysis as described below.

Precipitation of proteins in the brain and hemolymph samples was done by addition of 100 *μ*L of a 2% zinc sulfate in 50% methanol solution to a final volume of 150 *μ*L. The brain tissue was homogenized with an ultrasonic probe (Bandelin Electronic, Berlin, Germany) for 8 sec at a power of 19%. After homogenization all samples were centrifuged at 10 000*g* for 5 min at 4°C and 100 *μ*L of the supernatant transferred to Crimp *μ*-Vials (ScantecLab, Göteborg, Sweden) for analysis.

### Ex vivo experiments

**Figure 1:**
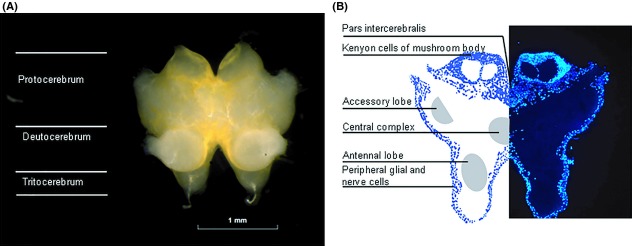
Anatomy of locust brain. The locust brain comprises three pairs of ganglia that lie above the esophagus. (A) A dissected *Schistocerca gregaria* brain, prepared for analysis, showing the bilobed brain consisting of the protocerebrum, deutocerebrum, and tritocerebrum. The protocerebrum receives sensory information from the compound eyes and the ocelli while deutocerebrum is a center for olfactory signal input. Tritocerebrum connects the insect brain via its circumesophageal connectives to the ventral nerve cord. (B) Section through the *S. gregaria* brain showing the typical peripheral localization of nucleated cells in insects (glia and neuronal cells). Shaded areas show some main regions of organized neuropil (for further information on the desert locust brain anatomy, see el Jundi et al. 2010).

The brain was dissected from the head as described above. The three merged ganglia of the brain (protocerebrum, deutocerebrum, and tritocerebrum) were prepared using fine forceps and the connections to the compound eyes were removed (Fig. 1). The brain was dissected free from fat and exposed to the test compounds in a microwell plate (96U Microwell Plates; Sigma-Aldrich, Stockholm, Sweden) containing 250 *μ*L of the test solution heated to 30°C in a block thermostat (HLC Technologies, Pforzheim, Germany). During model development the accuracy of the dissection procedure, to avoid affecting the tightness of the brain barrier, was repeatedly monitored using atenolol as a control. After a standard exposure time of 5 min the brains were removed from the wells and washed twice in ice-cold insect buffer. Following removal of the neural lamella (the outer connective tissue layer), the brains were transferred to 1.5 mL microcentrifuge tubes (Eppendorf) containing 50 *μ*L ice-cold insect buffer. Protein precipitation and brain tissue homogenization were performed as described above.

### Histology

Locust brains were fixed in 4% phosphate-buffered paraformaldehyde for 8 h and then washed in PBS and frozen at −18°C. The fixed and frozen brains were cut in 10 *μ*m sections, the nuclei stained with bisbenzimide and the slides photographed in an Olympus fluorescence microscope. Some brains were incubated in insect buffers containing red fluorescent 100 nm polystyrene nanoparticles for 15 min before fixation to stain the neural lamella outside the barrier layer.

### Quantitative analysis of drugs in hemolymph and brain tissue

Supernatant from the brain homogenates was analyzed by liquid chromatography-mass spectometry (LC-MS) using an Agilent 1200 HPLC coupled to a MSD 1100 detector (Agilent Technologies, Walbronn, Germany). The chromatographic column was a Phenomenex Kinetex (Værløse, Denmark), 50 × 4.6 mm with 2.6 *μ*m particles and was kept at 30°C. The mobile phase A was 0.1% formic acid and mobile phase B was 0.1% formic acid in methanol and analysis was performed using gradient elution from 5% to 90% mobile phase B. Detection was performed in SIM mode using the appropriate [M+1]^+^ ions in positive mode. Calibration standard solutions were prepared of the respective analytes in a similar way as the brain homogenates using insect buffer and the zinc sulfate reagent containing the internal standard. Quantities of 3-*μ*L aliquot of the samples and the calibration standards were injected into the LC-MS system. The lower limit of quantification (LLOQ) was typically in the range of 2 nmol/L in the final sample solution.

The measured concentrations in brain and hemolymph are the total compound concentrations, that is free compound plus compound bound to brain tissue and free compound plus compound bound to hemolymph proteins, respectively. This is in analogy to the corresponding brain:plasma ratio measurements often performed in mammalian studies (Hammarlund-Udenaes et al. 2008).

### Identification of locust Pgp-like protein-encoding transcripts

The human Pgp and the *Dm* Mdr65 protein sequences were used as queries for a tblastn search (Altschul et al. 1997) in the *Sg* transcriptome sequence data (unpubl. data from the research group of J. Vanden Broeck). Only those hits showing an e-value of 0.0 (excellent tblastn matches) for both queries were retained. The transcript sequences that met this criterion were subsequently retrieved from the database and verified by a reciprocal blastx search in the nr protein database of the National Center of Biotechnology Information (NCBI), restricted to *Homo sapiens* and *Dm* sequence data. The transcript sequences were translated by means of the ExPASy Translation Tool (http://www.expasy.org/tools/). The obtained *Sg* protein sequences, together with the human Pgp and *Dm* Mdr65 protein sequences were used as input for the Clustal Omega alignment algorithm (Goujon et al. 2010; Sievers et al. 2011).

### Statistical analysis

All values are expressed as mean ± standard deviation (SD). To determine statistical significantly differences among the experimental groups, the single-tailed Student’s *t*-test was used. A *P* value of <0.05 was deemed significant.

## Results

### In vivo brain uptake

**Figure 2:**
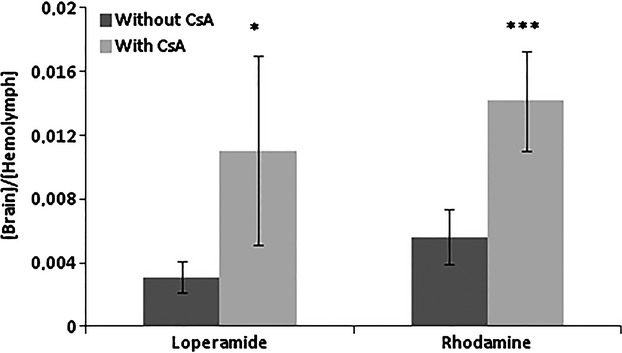
Effect of Pgp on locust brain drug distribution in vivo. Brain:hemolymph ratios of loperamide and rhodamine 123 following injection of the compounds into the hemolymph with (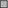) or without (▪) co-administered Cyclosporine A. Data presented as mean ± SD, *n* = 6 samples.(*P < 0.05, ***P < 0.001).

Previously it has been shown that the *Dm* brain concentration of Rho123 increases when the compound is co-injected with the Pgp inhibitor CsA (Mayer et al. 2009). To confirm this observation in *Sg* we have used the same study protocol to document the uptake of Rho123 in the locust brain. We also included the Pgp substrate loperamide in the in vivo study since it is taken up in the human brain when the Pgp efflux mechanism is inhibited (Elkiweri et al. 2009). The test compounds were injected alone or co-injected with CsA into the abdominal hemolymph. 15 min after injection hemolymph samples were collected and the brains were dissected for quantitative determination of test compound concentration. While there was no difference in hemolymph Rho123 or loperamide concentrations when the compounds were injected alone or co-injected with CsA (data not shown), there was a significant increase in brain uptake of both compounds when co-injected with CsA (Fig. 2).The utility of the in vivo locust model for screening and selection of potential CNS drugs could be challenged by the obvious complication of a rapid clearance from the hemolymph of the test drug and hence a drug-specific decrease in the exposure concentration. By using repeated hemolymph sampling (10 *μ*L) from the individual locusts we indeed found significant differences in clearance rates between loperamide and quinidine, two Pgp substrates, and between atenolol and propanolol, two compounds with similar molecular structures (Table 1).

**Table 1:** Drug elimination constants in locust calculated from *C* = *C*_o_*e*^−*kt*^

	Elimination (h^−1^)	SD
Atenolol	−1.46	0.14
Propranolol	−0.98^1^	0.10
Loperamide	−1.15	0.09
Quinidine	−0.70^2^	0.14

Administration by injection of 20 *μ*L of 20 *μ*mol/kg dose. From each animal, 10 *μ*L samples were collected at 0.08, 0.25, 0.75, 2, and 6 h after administration (*n* = 6).

1 *P* < 0.0001 atenolol versus propranolol.

2 *P* < 0.0001 loperamide versus quinidine.

### Ex vivo brain uptake

**Figure 3:**
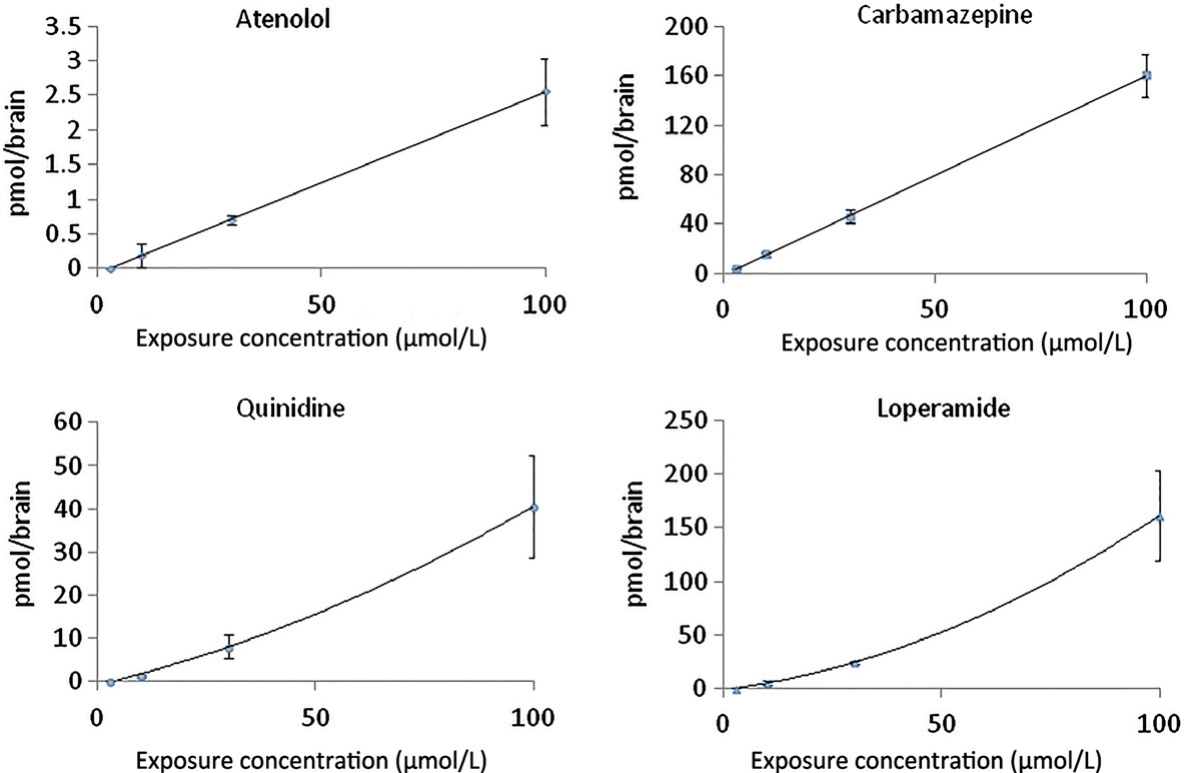
Locust brain drug uptake, concentration-dependence. Ex vivo brain uptake of atenolol, carbamezepine, quinidine, and loperamide following exposure at a concentration of 3, 10, 30, and 100 *μ*mol/L for 5 min. Data presented as mean ± SD, *n* = 3 samples in each group.

**Figure 4:**
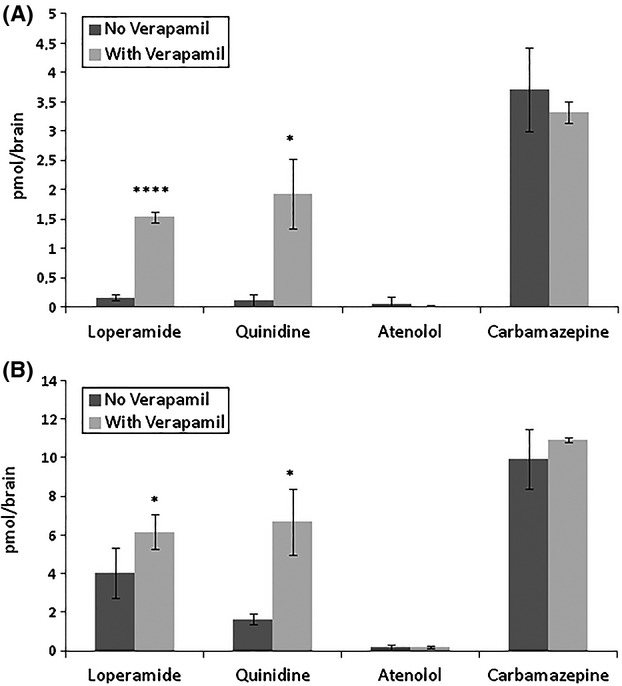
Effect of Pgp on drug concentration in locust brain. Ex vivo brain concentration of atenolol, carbamezepine, loperamide, and quinidine after 5 min exposure at concentrations of 3 *μ*mol/L (Fig. 3A) and 10 *μ*mol/L (Fig. 3B) with and without verapamil (30 *μ*mol/L). Data presented as mean ± SD, *n* = 3 samples in each group. (*P < 0.05, ****P < 0.0001).

To provide the drug discovery process with a relevant BBB permeability screening model, based on an in vivo like “whole-brain principle” and not affected by the hemolymph clearance of the test compound, we established an ex vivo locust brain barrier model. In this model, isolated locust brains are dissected from the insect and placed in a test tube (or well) containing the test compound dissolved in an insect buffer. In this setup, the exposure concentration and temperature is constant during the entire experiment. To validate the ex vivo model using drugs with well-known permeability characteristics we initially studied the concentration-dependent uptake of Cbz and atenolol, two compounds with markedly different BBB permeability. As is shown in Figure 3, brain uptake of the compounds in the ex vivo model was markedly different but both compounds displayed a linear increase in the brain uptake, strictly correlated to the exposure concentration, indicating that both compounds, as expected, are permeating the brain barrier by passive diffusion. When the ex vivo locust brains were exposed to increasing concentrations of the well-known Pgp substrates, quinidine and loperamide, the brain uptake pattern was markedly different from that of the passively diffusing compounds. The brain uptake increased more than linearly (Fig. 3). Differences in the concentration-dependent brain uptake profile of the vertebrate Pgp and the non-Pgp substrates were further investigated by studying the uptake of the test compounds at 3 and 10 *μ*mol/L exposure concentrations, with and without co-exposure to a Pgp transporter inhibitor. Since CsA has poor solubility it is not useful in the locust ex vivo model where an inhibitor concentration of 25 *μ*mol/L is used. Instead we used verapamil, a well-studied Pgp inhibitor, which is used routinely in in vitro models. In Figure 4 it is seen that Cbz and atenolol uptake were unaffected by co-exposure to verapamil while verapamil significantly increased the brain concentration of quinidine and loperamide. These observations strongly suggest that the locust ex vivo model exposed to the vertebrate Pgp transporter inhibitor verapamil can be used to discriminate between passively diffusing test compounds and Pgp transporter substrates.

### Investigation of energy-dependent efflux

**Figure 5:**
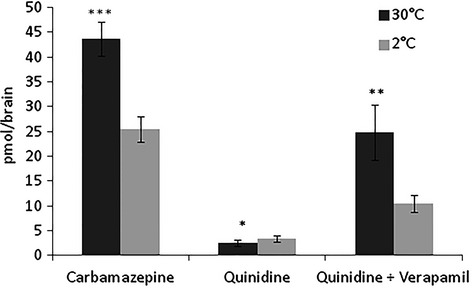
Effect of temperature on drug concentration in locust brain. Brain concentration of carbamazepine, quinidine, and quinidine + verapamil (30 *μ*mol/L) after ex vivo exposure at a concentration of 3 *μ*mol/L and at the temperatures 30°C or 2°C for 5 min. Data presented as mean ± SD, *n* = 3 samples in each group. (*P < 0.05, **P < 0.01, ***P < 0.001).

Since Pgp-mediated transport is strictly ATP- and temperature-dependent it should be possible to differentiate a passively diffusing compound such as Cbz from an actively effluxed Pgp substrate such as quinidine by comparing the locust brain uptake at different temperatures. Accordingly, it was found that the passive diffusion of Cbz into the ex vivo locust brain was markedly increased when the exposure temperature was raised from 2°C to 30°C (Fig. 5). A corresponding increase in quinidine uptake was not seen indicating a compensatory and temperature-dependent increase in Pgp efflux transport efficiency. This notion was confirmed when the Pgp transporter was inhibited by verapamil. Here the Pgp substrate quinidine showed temperature-dependent brain permeation similar to that for the passively diffusing compound Cbz. These observations strongly indicate that the difference in brain uptake between Pgp and non-Pgp substrates in the locust model depends on an active energy- requiring efflux mechanism similar to vertebrate Pgp efflux.

### Identification of locust Pgp-like protein-encoding transcripts

**Figure 6:**
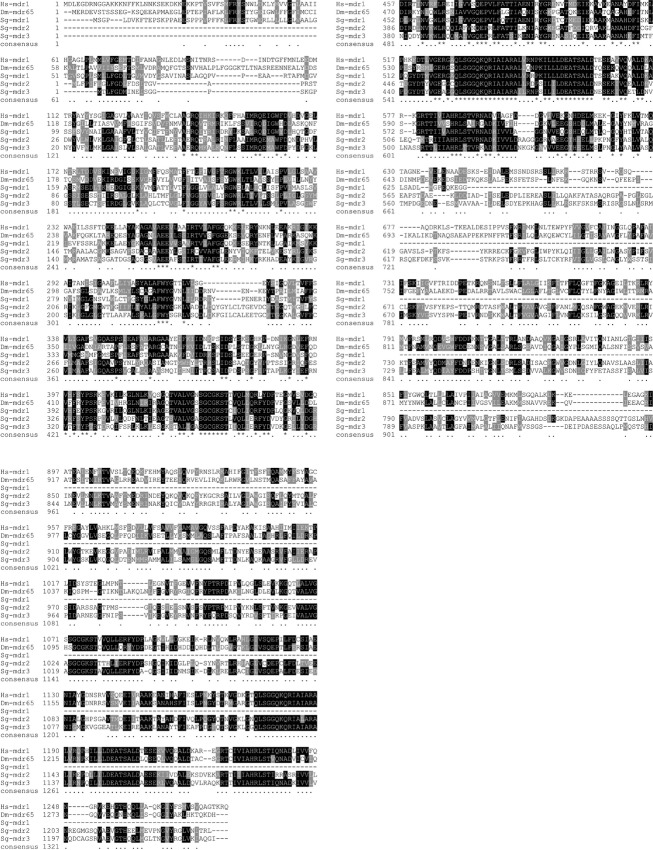
Comparison of Pgp protein sequence in human, *Drosophila,* and locust. Multiple sequence alignment of the human Pgp (Hs-mdr1), the *Dm* Mdr65 (Dm-mdr65), and the three *Sg* Pgp/Mdr65 orthologs (Sg-mdr1-3). The protein sequence alignment was performed by Clustal Omega analysis, available from the European Bioinformatics Institute (Goujon et al. 2010; Sievers et al. 2011). All parameters were set at default values. The BoxShade algorithm (http://www.ch.embnet.org/software/BOX_form.html) was used for analysis of identical and similar amino acid residues. Highly conserved residues occurring in at least 60% of the sequences are highlighted in black. Residues similar in chemical nature occurring in at least 60% of the sequences are highlighted in gray. The consensus line includes the following symbols: “*” representing a particular position at which an amino acid is conserved in all sequences, “.” representing a position for which at least 60% of the amino acid residues are identical or similar. (The Sg-mdr1-encoding transcript sequence was only partially retrieved from the available *Schistocerca gregaria* transcriptome database.)

Investigation of the sequence similarity between *Sg*, *Dm*, and human Pgp was made by a tblastn search in the *Sg* transcriptome data. This search revealed three transcript sequences showing an e-value of 0.0 for both the human Pgp and the *Dm* Mdr65 queries (Fig. 6). The fact that these transcripts indeed encode Pgp/Mdr65 orthologs was confirmed by the reciprocal blastx search in the NCBI nr protein database. One of the transcripts appeared to be represented in the database only as a partial sequence, since it does not contain the complete open reading frame. The protein sequences corresponding to the three transcripts were termed *Sg*-Mdr1, *Sg*-Mdr2, and *Sg*-Mdr3, and they show, respectively, 24.5% (partial sequence), 36.2%, and 37.2% sequence identity with the human Pgp, and 22.6% (partial sequence), 32.2%, and 33.1% identity with the *Dm* Mdr65. The human Pgp and *Dm* Mdr65 display 41.4% sequence identity. Like the human Pgp and the *Dm* Mdr65, the *Sg*-Mdr2 and *Sg*-Mdr3 sequences contain two conserved ABC transporter transmembrane domains (http://pfam.sanger.ac.uk/: pfam00664), as well as two conserved ABC domains (NCBI Conserved Domains database: cd03249). The partial *Sg*-Mdr1 sequence displays one of each domain, corresponding to the first one present in each of the other sequences.

### Localization of insect brain cells

**Figure 7:**
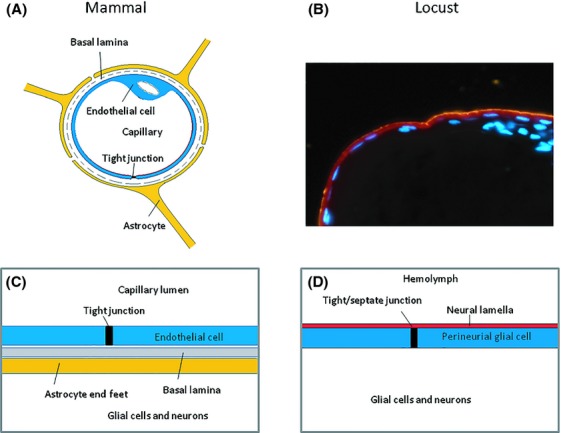
Comparison of the mammalian and locust brain barrier. (A) Cross section through a mammalian brain capillary showing a vascular endothelial cell with tight junction, the site of the blood-brain barrier. Underlying the endothelium is a basal lamina, which is surrounded by the endfeet of the astrocytic glial cells. (B) Bisbezimid-stained histological section through the tritocerebral part of a locust (*Locusta migratoria*) brain showing the elongated nuclei of perineurial glial cells and nuclei of peripheral cortex glial cells and nerve cell bodies. Outside the perineurium brain barrier is a neural lamella, stained with fluorescent polystyrene 100 nm nanoparticles (red). (C) Schematic drawing of the mammalian blood-brain barrier components. To reach its target on a neuronal cell a therapeutic compound in the capillary blood has to cross the endothelial cell layer, the basal lamina, and the astrocyte layer. (D) In the insect a compound in the hemolymph surrounding the brain has to cross the neural lamella, the perineurium brain barrier, and in some regions also layers of subperineurium glial cells to reach the brain neurons. Note the marked difference in number of nucleated cells in the tritocerebral peripheral brain cortex between *Locusta migratoria* shown here and *Schistocerca gregaria* (Fig. 1), used in this study. Therefore, in our proposed model, the locust brain barrier appears to consist of a single layer of elongated nucleated glial cells.

Histological sections through the brain of both the desert locust (*S. gregaria*) (Fig. 1B) and the migratory locust (*Locusta migratoria*) (Fig. 7B) showed the typical localization of glial and neuronal cell bodies to the peripheral cortex while the central region of the brain contains neuropil and tracts (Fig. 1B). In some areas of *L. migratoria* brain we found only a single layer of cell bodies (Fig. 7B). The glial cells of this layer are characterized by elongated nuclei.

## Discussion

Compared to the in vivo mouse Pgp KO model the majority of the developed in vitro screening models do not reach sufficient accuracy in differentiating Pgp from non-Pgp substrates (Mahar Doan et al. 2002; Feng et al. 2008; Liu et al. 2008). Recently, zebrafish and insects have attracted attention for their utility in studies of the vertebrate BBB (Xie et al. 2010; Geldenhuys et al. 2012) and insects have been shown to be suitable models for vertebrate BBB permeability prediction in early drug discovery (Nielsen et al. 2011; Andersson et al. 2013). These models make use of the evolutionarily conserved essential mechanisms important in CNS protection, which operate in mammals and nonvertebrate organisms (Bundgaard and Abbott 2008; DeSalvo et al. 2011). Despite marked differences in brain structure between insects and vertebrates there are many common features at the cellular level of their brain barriers. In both insects and vertebrates there is one specific cell layer making up the barrier function. We have adopted the terminology from earlier studies in the locust that identified glial cells with elongated nuclei (Fig. 7B) as constituting the perineurium and function as the brain barrier (Gocht et al. 2009). Recently the understanding of the anatomy of the insect brain barrier has expanded considerably from studies in *Drosophila* (DeSalvo et al. 2011). These studies demonstrate the presence of two subtypes of surface glia at the *Drosophila* brain barrier, the perineurial and the subperineurial glia, which both appear to form complete layers of ensheathing glial cells that surround the brain. The flattened stellate morphology of the perineurial glia may be a reason for not observing the presence of this glial layer in the locust light microscopic sections and therefore it may be suggested that the labeling of the surface perineurial glia in this study (Fig. 7) corresponds to the subperineurial glia sheath in *Drosophila* (DeSalvo et al. 2011). This is the principal cell layer of the brain chemical protection, and the laterally localized junctional barriers to paracellular diffusion are made up of nearly identical and homologous proteins in vertebrates and insects (Fig. 7) (Wu and Beitel 2004; Danemann and Barres 2005; Banerjee and Bhat 2006; Stork et al. 2008).

Demonstration of homologies and functional parallels between insect and vertebrate Pgp efflux transporters has been shown where the *Dm* Mdr65 transporter was characterized by exhibiting 42% sequence identity to the mammalian ABC transporter MDR1/Pgp, being localized at the periphery of the glia:hemolymph barrier and showing Pgp efflux properties in Mdr65-transfected human embryonic kidney (HEK) cells (Mayer et al. 2009). These observations strongly indicate the homologies between the insect and mammalian ABC efflux transporters based on studies on the fly. The transcriptome data presented here also indicate the homologies between insects (locusts) and mammals including human.

In this study it was shown that in vivo inhibition of the Pgp transporter in the locust by CsA resulted in a significantly increased brain uptake of both Rho123 and loperamide, two well-known Pgp substrates. These data strongly suggest that the locusts have transporter proteins present in the brain barrier and that these efflux proteins can be inhibited by CsA as shown in the *Dm* model (Mayer et al. 2009). Importantly, locust transcriptome data show a high degree of identity of the *Sg*-Mdr1-3 protein sequences to the human Pgp sequence and conserved domains indicating that these locust proteins are true Pgp orthologs, which may correspond to the brain transporter proteins inhibited by CsA. In addition to the Pgp transporter homologies, the locust has the advantage of being a large insect with a large-sized brain.

Screening of potential vertebrate Pgp transporter substrates can be done successfully in transgenic and WT mice in vivo but these models do not show sufficient cost efficiency to be used as screening models. Therefore, sophisticated mammalian in vitro co-culture models have been developed demonstrating functional expression of Pgp (Gaillard et al. 2000). However, even these models are unable to replicate all the physiological functions of a dynamic brain barrier (Abbott et al. 2010; DeSalvo et al. 2011) and they suffer from complex culture technology (Mensch et al. 2009). As shown in this study the locust in vivo model has all the endogenous brain barrier features and exhibits a relevant structure and function of its brain barrier. However, this study also shows that intrahemolymphic injected test compounds are subject to a rapid and differential elimination from the hemolymph. Thus, the brain exposure of the compound is affected by drug-related elimination over time requiring complex AUC (area under curve) calculations in order to estimate the brain-to-plasma ratio. Since the aim with the current model development was to establish a cost efficient model, which could be used in drug discovery, we focused on an ex vivo approach. In this model, the whole brain is dissected out and exposed at a constant temperature and substrate concentration. In contrast to both vertebrate and the locust in vivo models the locust ex vivo model is not affected by a time-dependent decrease in exposure concentration. By using the whole brain during the exposure procedure this model represents a significant step toward a model retaining the relevant in vivo biology of a dynamic and physiologically functioning brain barrier. In the locust models housed animals of appropriate ages can be used. This, together with the simplicity by which the brain is dissected out and incubated makes the locust ex vivo model easy to handle and it does not require a complex culture technology typical of recently developed in vitro models.As expected, the uptake of the low and high permeability compounds, atenolol and Cbz, respectively, showed a dose-related linearity in brain uptake in the ex vivo model. The locust ex vivo model brain uptake confirmed the difference seen in vertebrate barrier permeability for the two test compounds, and confirmed the high dynamic range of the locust model (ratio of permeabilities for these two compounds is ∼64). By contrast, uptake of the Pgp substrates (loperamide and quinidine) increased more at higher concentrations, indicating saturation of the Pgp transporter, that is uptake by diffusion was able to override efflux by the transporter. Furthermore, the ex vivo model showed that co-treatment with the vertebrate Pgp transporter inhibitor verapamil significantly increased the brain uptake of the two Pgp substrates. The data showed that low permeability compounds such as atenolol can be discriminated from Pgp substrates such as quinidine by using co-exposure with verapamil. For model comparison, the quantitative effect of verapamil is similar to the effect found in a mammalian co-culture model (Culot et al. 2008). These findings support the evidence that the verapamil-promoted uptake of the Pgp substrates in the locust is due to the presence of an efflux transporter that is efficiently inhibited by the Pgp inhibitor verapamil.The present and recently reported data (Andersson et al. 2013) show a remarkable similarity between locust ex vivo and mouse Pgp KO/WT data (Doran et al. 2005) confirming the accuracy of the locust model in differentiating Pgp from non-Pgp substrates. Furthermore, both models showed that CNS (e.g., risperidone and citalopram) and non-CNS drugs (e.g., loperamide and quinidine) are affected by Pgp transporter efflux, confirming that CNS drugs could also be Pgp substrates. In addition to the Pgp efflux transporter, the breast cancer resistance protein (BCRP or ABCG2) is localized to the brain of human and mice and found to be a major efflux transporter at the BBB (Ohtsuki and Terasaki 2007). In fact, studies on isolated brain capillary endothelial cells showed that the protein expression of BCRP was almost two times greater in humans than in mice while the expression level of Pgp in humans was about half of that in mice (Uchida et al. 2011). A recent study of the sequence similarities between the protein sequence corresponding to the locust *Unigene18039* encoded transcript and the human ABCG2 transporter protein BCRP), showed a 40% identity (Al-qadi, poster presented at the *16th International Symposium (2013) on “Signalling in the Blood-Brain Barriers,”* Sumeg, Hungary). The presence of a non-Pgp efflux transporter was also indicated by the observation of a marked, more than dose-related, brain uptake of Bupropion, a BRCP transporter substrate, in the locust ex vivo model after increased exposure concentrations, while the uptake was unaffected by treatment with the Pgp inhibitor verapamil (Andersson et al. 2013). These observations may indicate both the presence and physiological function of a BCRP transporter homologue in the locust brain barrier. Despite the common characteristics of locust and mice in identifying Pgp substrates (Andersson et al. 2013), the modest sequence identity between the locust and human Pgp (and likely also BCRP) transporters, indicates potential species differences in the interaction of specific drugs with the transporters.The ABC – superfamily efflux transporters play a central role in protecting the brain from toxic xenobiotics, which also affect the uptake and distribution of drugs to their brain tissue targets. The less well understood complex network and interactions between these transporters (Sharom 2008) may warrant the use of a simplified model indicating whether a test compound is effluxed by a ATP- binding cassette transporter or not.

Since it is well-known that the Pgp efflux mechanism in vertebrates is an active process requiring ATP (Li et al. 2010) it was expected that a reduction in the exposure temperature should reduce both the permeation of passively diffusing compounds and the efficiency of the efflux transporters also in the insect model. Indeed, this study confirmed the utility of a locust ex vivo model where brain uptake profile at normal and low (energy uncoupling) temperature was markedly different for a passively diffusing compound, Cbz, and a Pgp efflux transported compound, quinidine. This model may, therefore, play a role as a first line in the identification of non-specific efflux transporter substrates of compounds in early drug discovery.

To obtain an experimental BBB model with no disturbance to all the necessary signaling interactions at the barrier (Danemann and Barres 2005), an optimal model would be to use an intact brain, exhibiting a natural, undisturbed, dynamic brain barrier. The locust ex vivo model fulfills these criteria and showed high reproducibility, which hence could be useful in compound screening in the early drug discovery phase. Importantly in this model there is no choroid plexus or CSF, hence the brain barrier described is the only layer governing CNS drug distribution; this simplifies analysis compared to the mammalian brain. The ex vivo model approach is supported by the recently published comprehensive studies on *Dm* (Stork et al. 2008; DeSalvo et al. 2011). Despite the species differences between insects and human, the model exhibits strong evolutionary conservatism with respect to the Pgp efflux transporter, making the model a reliable tool in early drug screening of potential Pgp substrates based on quantitative measurements of brain drug uptake.

## Abbreviations

ABC: ATP-binding cassette
AUC: area under curve
B/P: brain:plasma ratio
BBB: blood–brain barrier
BCRP: breast cancer-resistant proteins
Cbz: carbamazepine
CNS: central nervous system
CsA: cyclosporin A
KO: knockout
LLOQ: lower limit of quantification
MDCK: Madin-Darby canine kidney
MDR1-MDCK: MDR1-transfected MDCK
MDR1: multidrug resistance protein-1
NCBI: National Center of Biotechnology Information
Pgp: P-glycoprotein
Rho123: rhodamine 123
SD: standard deviation
WT: wild type
